# How musical experience affects tone perception efficiency by musicians of tonal and non-tonal speakers?

**DOI:** 10.1371/journal.pone.0232514

**Published:** 2020-05-08

**Authors:** Si Chen, Yiqing Zhu, Ratree Wayland, Yike Yang

**Affiliations:** 1 Department of Chinese and Bilingual Studies, The Hong Kong Polytechnic University, Kowloon, Hong Kong SAR, China; 2 Hong Kong Polytechnic University-Peking University Research Centre on Chinese Linguistics, Hong Kong, China; 3 Department of Linguistics, University of Florida, Gainesville, FL, United States of America; University College London, UNITED KINGDOM

## Abstract

**Purpose:**

To investigate if, regardless of language background (tonal or non-tonal), musicians may show stronger CP than non-musicians; To examine if native speakers of English (English or non-tonal musicians henceforth) or Mandarin Chinese (Mandarin or tonal musicians henceforth) can better accommodate multiple functions of the same acoustic cue and if musicians’ sensitivity to pitch of lexical tones comes at the cost of slower processing.

**Method:**

English and Mandarin Musicians and non-musicians performed a categorical identification and a discrimination task on rising and falling continua of fundamental frequency on two vowels with 9 duration values.

**Results:**

Non-tonal musicians exhibited significantly stronger categorical perception of pitch contour than non-tonal non-musicians. However, tonal musicians did not consistently perceive the two types of pitch directions more categorically than tonal non-musicians. Both tonal and non-tonal musicians also benefited more from increasing stimulus duration in processing pitch changes than non-musicians and they generally require less time for pitch processing. Musicians were also more sensitive to intrinsic F0 in pitch perception and differences of pitch types.

**Conclusion:**

The effect of musical training strengthens categorical perception more consistently in non-tonal speakers than tonal speakers. Overall, musicians benefit more from increased stimulus duration, due perhaps to their greater sensitivity to temporal information, thus allowing them to be better at forming a more robust auditory representation and matching sounds to internalized memory templates. Musicians also attended more to acoustic details such as intrinsic F0 and pitch types in pitch processing, and yet, overall, their categorization of pitch was not compromised by traces of these acoustic details from their auditory short-term working memory. These findings may lead to a better understanding of pitch perception deficits in special populations, particularly among individuals diagnosed with autism spectrum disorder (ASD).

## Introduction

The link between music and language has been a subject of great interest among researchers from various disciplines, and evidence suggesting a connection between musical abilities and phonetic processing skills is growing [1]. Acoustic fundamental frequency (F0) or its auditory impression of pitch is a common perceptual attribute in music and language. It differentiates notes in music and word meaning in lexical tone languages such as Mandarin, Thai and Vietnamese. Associations between musical ability and accuracy at perceiving lexical tone contrasts have been reported in previous investigations [1–11]. Specifically, besides being more auditorily sensitive to pitch differences in general, these studies suggest that musicians perform better than non-musicians in identifying and discriminating lexical tones. Perceptual skills that lead to musicians’ superior ability to process and encode acoustic information in lexical tones have also been investigated. For instance, it is found that brainstem encoding of Mandarin Tones 1, 2 and 3 were more faithful and robust among amateur musicians than non-musicians despite not having prior exposure to Mandarin [12]. This finding is intriguing in so far as it is consistent with what is reported for native Mandarin listeners who have extensive exposure to lexical pitch [13]. In this study, Krishnan et al found robust encoding at the brain stem level for all four Mandarin tones among native Mandarin listeners. According to [12], these results provide “neurophysiological explanations for musicians’ higher language-learning ability” (p. 420). However, according to [14], even though brainstem representation of musical pitches among both Mandarin listeners and English-speaking musicians are stronger in comparison to English-speaking-non-musicians, neither Mandarin listeners nor non-musicians were as accurate as musicians in discriminating musical pitch in behavioral tasks. From these results, the authors inferred that “sensory-level enhancement of musical pitch information yields cognitive-level perceptual benefits only when that information is behaviorally relevant to the listener” (p. 1). It is conjectured that musical training may improve representations of auditory objects, network control and mapping of sounds to memory templates, potentially leading to musicians’ advantage in categorical perception of speech [15].

Besides F0, other acoustic dimensions including amplitude, duration and phonation type may also aid lexical tone perception [16]. Duration, amplitude [17–21] and phonation types (i.e., creaky or glottalization; [4, 22, 23] have all been shown to play a role in lexical tone perception. Therefore, it is not clear if isolated use of pitch was responsible for advanced lexical tone perception ability among musicians reported in previous studies using naturally-produced lexical tone stimuli [7]. Furthermore, different F0 dimensions including average F0, F0 onset, F0 offset and F0 direction or contour (i.e., rising and falling) are used to distinguish different pairs of lexical tones. However, it has yet to be determined which of these F0 dimensions contributes to musicians’ enhanced lexical tone perception.

In addition, since individuals vary greatly in their pitch range, besides sensitivity to the acoustic differences, robust formation of lexical tone categories is also required for successful acquisition of lexical tone contrasts [7]. To accomplish this, listeners must differentiate within- versus between-category acoustic differences and learn to consistently ignore the former while maintaining sensitivity to the latter. This perceptual behavior is known as categorical perception.

### Categorical perception

Categorical perception (CP) is a perceptual phenomenon whereby listeners can discriminate stimuli if they are drawn from separate categories, but they discriminate the stimuli poorly from the same category. In other words, they are more sensitive to between- than within-category differences. Identification and discrimination tasks are both necessary to establish categorical perception of a continuum. The identification task shows how the stimuli are categorized into different categories, whereas the discrimination task reveals how well members of the same or different categories are discriminated. The presence of CP is indicated by series of characteristics such as sharp category boundary [24]. The strict version of CP holds that identification completely predicts discrimination. Empirically, however, listeners are often better at discriminating within-category stimuli than predicted by their identification. In the auditory domain, CP of segments (i.e., vowels and consonants) have been relatively well studied compared to CP of a suprasegment such as lexical tones.

The strength of CP varies as a function of stimuli. In comparison to vowels and fricatives, perception of stop consonants (e.g. stops with different voice onset time) are more categorial, exhibiting sharp category boundaries and discrimination patterns accurately predicted by categorization [25–27]. On the other hand, identification functions for vowels and fricatives are less steep [28–30], and listeners are not simply relying on category labels for discrimination [31].

Several theories have been proposed to account for CP. A dual process has been proposed in speech processing [32]. According to this model, two types of memories: phonetic and auditory short-term memories, are implicated in speech perception. Phonetic short-term memory is involved with the categorization process, while auditory short-term memory is associated with the discrimination process. In other words, listeners’ discrimination of two different phonetic segments is based on whether or not they categorize the two stimuli as belonging to the same or different phonetic categories on the basis of phonetic features represented in phonetic short-term memory. On the other hand, discrimination between two identical phonetic segments is based on a comparison of stored auditory information or traces of the acoustic features of the stimuli represented in their auditory short-term memory. Both memories are activated during speech processing and CP is mainly determined by the degree to which auditory short-term memory is employed in discrimination [33]. To Pisoni, these results suggested that auditory and phonetic memory codes are distinct and that auditory short-term memory for consonants is different from that for vowels. Specifically, the acoustic information needed to discriminate two physically different stop consonants from the same phonetic categories is not as well represented as that of vowels in auditory short-term memory. One possible explanation is that the critical acoustical cues for stop consonant distinction (e.g., formant transitions, voice-onset-time) are relatively shorter than those for vowels (e.g., steady-steady state formant frequencies) and, thus, are not strongly coded in auditory short-term memory. This is known as the cue-duration hypothesis [29].

Auditory distinctiveness is another explanation proposed by some investigators to account for differences between categorization and discrimination patterns among vowels and consonants [34]. Auditory distinctiveness is associated with perceptual range, defined as the sum of *d’* between adjacent stimuli, which is greater for vowels than for consonants; therefore, vowels are more discriminable than consonants.

It is proposed that differences observed in categorizing and discriminating stop consonants and steady-state vowels arise from general differences in how changing and steady acoustic cues are processed by the auditory system in general [35]. They found that listeners’ categorization of stimuli varied based on the changing cue. This finding is similar to what is reported for consonants, defined by rapidly changing cues, and for dynamic vowels, defined by both steady-state and rapidly changing acoustic cues. However, similar to vowel perception defined by stead-state cues only, they found that listeners’ discrimination of steady-state non-speech stimuli is more accurate than their categorization. More recently, contingent categorization has been proposed that the categorization of acoustic cues is also affected by context information [36, 37]

### Categorical perception of lexical tones

For lexical tones, a few studies showed that native speakers perceived Mandarin tones categorically by native listeners, but non-native tone language listeners did not [38–41] However, a recent study [42] showed that, similar to native listeners, English-speaking advanced learners of Mandarin Chinese exhibited categorical perception of Mandarin tones. These results suggest that CP is affected by experience and can be learned. The finding that native Thai listeners showed stronger categorization of speech tone continua than English listeners, but are similar to English listeners in performance when perceiving non-speech continua further suggests the role of experience (with lexical tone in this case) with CP [43]. In addition, due to their lack of experience with lexical tones, non-native tone listeners tend to perceive a tone continuum pyschoacoustically. For example, in contrast to Taiwan Mandarin listeners whose tone perception is quasi-categorical, French listeners’ perception seems to be more psychophysically based rather than linguistically determined [38]. Similarly, different from Mandarin and Cantonese listeners speaking a tonal language, German listeners exhibited broader identification boundary widths and discrimination boundaries that are psychophysically rather than linguistically determined [40].

Interestingly, CP was found for some lexical tones but not others. For example, CP was found for a high-rising to high-level tone continuum in Cantonese [44] and a rising to level tone continuum in Mandarin [45]. On the other hand, no evidence of CP was found for level tones in Thai or Cantonese [44, 46]. The degree of CP also differs for different tones. For both tonal and non-tonal listeners, categorization is more prominent for the falling contours than the rising ones [47].

Fewer studies investigated CP of pitch stimuli among musicians. Categorization of synthetic speech triads is more prominent, and the discrimination pattern is better predicted through categorization among musicians than among non-musicians [48]. Similarly, musical training sharpens categorical boundaries and improves discrimination of three-pure-tone sequences [49]. From their experiment, it can be concluded that musicians’ processing of pitch (sine-tone) intervals is categorical [50]. However, unlike Mandarin speakers, both English musicians’ and non-musicians’ perception of the Mandarin T2-T3 continuum was continuous [9]. Mandarin musicians and non-musicians showed similar sensitivity to lexical tones, and both of them exhibited reduced sensitivity for within-category tone pairs [10]. More recently, comparable identification functions with similar steepness and locations of categorical boundaries among Chinese musicians and non-musicians were found in their processing of Mandarin Tone 1-Tone 4 continuum [51]. However, musicians exhibited superior discrimination performance on within-category stimuli over non-musicians.

In sum, categorical perception demonstrates that previously formed phonemic categories affect speech perception. Within-category members are treated as equivalent and are, therefore, poorly discriminated, whereas between-category tokens are well discriminated. CP has been found to be stronger in consonants than in vowels. CP has also been found for lexical tones among native listeners and advanced learners, suggesting the role of exposure to CP. Due to their lack of experience with lexical tones, non-native listeners of tone continua are strongly pyschoacoustically based. Some CP studies involving musicians show that musical abilities enhance both categorization and within-category discrimination in both speech and non-speech stimuli, while others show comparable results between musicians and non-musicians.

### Vowel quality and stimulus duration in tone perception

The effects of intrinsic F0, type of pitch directions and stimulus duration on categorical perception of pitch contours among tonal (Mandarin) and non-tonal (American English) non-musicians have been investigated to determine minimum duration required to perceive rising and falling pitch contours [47]. While that study focused on the effects of these factors on the minimum duration needed for effective discrimination of pitch contour among non-musicians from a tonal and a non-tonal language [47], this current study focused on the influence of these factors on the categorization of pitch contour among musicians from both language types. The results shed light on the ability of musicians in accommodating factors such as vowel quality that may affect f0 and also the efficiency in tone perception.

**Fig 1:**
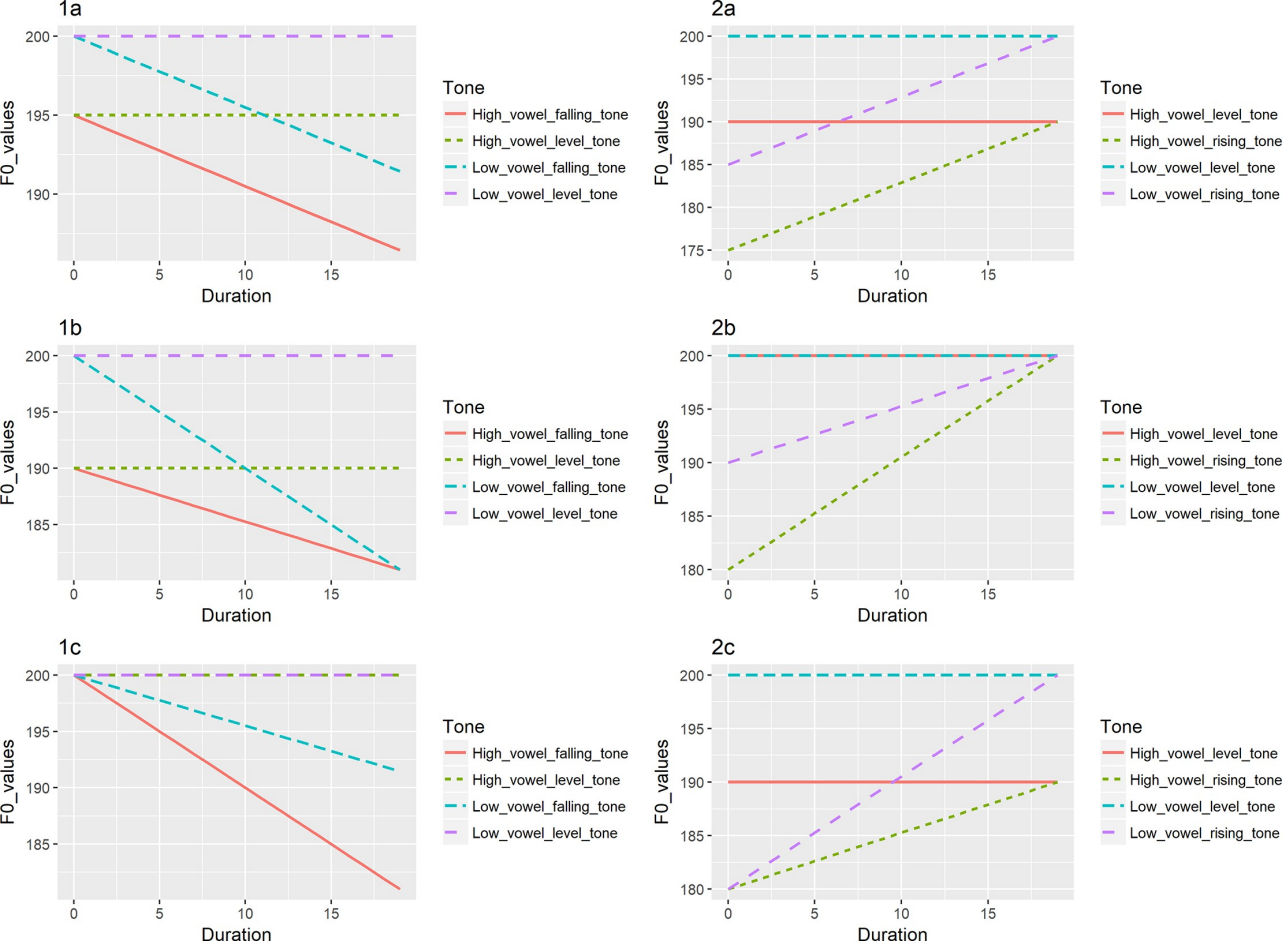
Hypothesized time required to identify rising or falling pitch directions. 1a) Same amount of time required to perceive falling tones on low and high vowels 1b) falling tones on high vowels requiring a long perception duration; 1c) falling tones on high vowels requiring a shorter duration to be perceived; 2a) Same amount of time required to perceive rising tones on low and high vowels; 2b) Rising tones on low vowels requiring a longer perception duration; 2c) Rising tones on high vowels requiring a longer perception duration.

The well-known intrinsic F0 effects refer to the fact that high vowels are usually correlated with higher F0 values and low vowels with lower F0 values in speech production cross-linguistically [52]. However, the opposite of this relationship has been observed in speech perception. When the pitch on low and high vowels is manipulated to be the same acoustically, low vowels are usually perceived to have a higher pitch than high vowels [53]. It has been shown that intrinsic F0 effects significantly contributed to CP among non-musicians, though the effect was relatively limited [47]. The current study examines intrinsic F0 effects on musicians’ categorical perception. Six hypothetical situations have been proposed to capture the role that intrinsic F0 effects may play in perception of rising and falling pitch directions [47]. They hypothesized that the time required to identify rising or falling pitch directions may differ on low vs. high vowels due to perceptual compensation of the intrinsic F0 effect. As in Fig 1, situations (1a) and (2a) depict scenarios where falling tones and rising tones produced on a high and a low vowel would require the same amount of time to be accurately perceived. In these cases, the F0 of the onset and offset of both tones are perceived as lower on a high vowel than on a low vowel to the same extent, leading to a similar perceived slope, and thus, a similar amount of time required to be perceived accurately. However, in situations (1b) and (2b), more time is required to perceive a falling tone on a high vowel than a low vowel due to a less steep slope on a high vowel since the offsets are perceived to be similar on high and low vowels, but the onset is perceived to be lower for high vowels. On the other hand, less time may be required to accurately perceive a rising tone on a high vowel due to a sharper slope. In contrast to situations (1b) and (2b), in situations (1c) and (2c), more time is required to perceive a falling pitch direction on low vowels than high vowels due to a less steep slope in the pitch direction on low vowels since the onsets are perceived to be similar on high and low vowels, but the offset is perceived to be lower for high vowels than low vowels. In contrast, less time should be required to perceive a rising pitch direction on low vowels due to a perceived steeper slope of the pitch direction.

Perception of a falling pitch contour required more time on low vowels (situation 1c) [47]. However, differentiating a rising pitch direction from a level one required a similar amount of time on low and high vowels (situation 2a).

Stimulus duration also plays a critical role in speech perception. In addition to the cue-duration hypothesis, which explains why consonants are perceived more categorically than vowels, it has been shown that shorter vowels are perceived more categorically than longer vowels [32]. Also, many studies have reported the interaction between tones and the perceived vowel duration. Usually, vowels bearing dynamic tones such as falling and rising tones are perceived to be longer than vowels bearing level tones [54, 55]. More recent studies demonstrated that the older population may perceive Mandarin tones less categorically in shorter duration [56] and stimulus duration facilitates categorical perception of pitch directions for both tonal and non-tonal younger adult speakers [47].

### The current study

We have three research goals for the current study: 1) to examine whether musicians are able to accommodate many factors affecting the same acoustic cue f0, whether they may better use longer stimuli for auditory representation and whether musicians show more differences in the time required to perceive the two pitch types; 2) to investigate if regardless language background (tonal or non-tonal), musicians show stronger CP than non-musicians across different durations; 3) and to examine if musicians need to slow pitch processing to achieve better identification and discrimination.

For the first aim, since f0 plays a critical role in lexical tone perception and it can signal vowel height at the same time, we aim to examine whether musicians may tease apart the various functions of f0 and compensate for the effects on f0 induced by vowel height. As mentioned earlier, a compensatory mechanism, where the relationship between vowel quality and f0 values is the opposite in speech perception compared to speech production, has been documented. In this study, we aim to examine whether musicians compensate more for intrinsic F0 effects in perception than non-musicians. It has been reported that listeners with higher musical ability were more likely to be able to separate acoustic cues such as spectral cues and f0 and use the most relevant acoustic cue f0 in pitch perception [57]. Those with lower musical ability tend to rely on both spectral and f0 cues. Since vowel height is correlated with f0 and f0, in turn, is an important cue of lexical tones, it is thus worth testing to see if, in addition to being able to differentially attend to different acoustic cues well, musicians also excel at accommodating or mitigating factors that may affect those same acoustic cues. Second, it remains to be investigated whether musicians are more efficient in making use of increased stimulus duration in pitch perception. Although it has been reported that musicians perform better than non-musicians in identifying and discriminating lexical tones [5], it remains unknown if their greater sensitivity to f0 comes at the cost of a lesser processing efficiency. By examining the effect of stimulus duration, we may explore whether musical training increases sensitivity to pitch of lexical tones at the cost of slower processing. Third, we aim to test whether there is a perceptual compensatory mechanism in perceiving different types of pitch directions (falling vs. rising) by musicians. In speech production, it is shown that rising pitch directions require longer time to produce [58]. We aim to test whether rising pitch directions also require longer time to perceive by musicians and whether musicians show more differences in the time required to perceive the two pitch types due to their sensitivity to pitch.

For the second aim, since no agreement has been reached regarding the effects of musical experience on categorical perception by tonal and non-tonal speakers, we conduct a more comprehensive study including tonal and non-tonal musicians, different types of pitch directions (rising vs. falling), various duration and vowel quality (low vs. high). All the characteristics of CP are examined for different stimulus duration values to gain a comprehensive picture of the effects of musical experience both in tonal and non-tonal speakers.

Regarding the third aim, we wonder if musicians would require a shorter time to accurately perceive pitch directions compared to non-musicians and if a ceiling effect would be found for tonal speakers, where musical experience cannot further improve the efficiency of pitch processing. We also aim to propose formulae of time required for above-chance identification accuracy level of pitch directions, and a ranking of four groups in the efficiency of pitch processing. In addition, we aim to compare the previously reported formulae for speech production [58] with speech perception for musicians.

## Methodology

### Subjects

Thirteen American English L1 musicians (English musicians henceforth) (7 males; 6 females; mean age ± SD: 23.38 ± 5.06 years) and thirteen Chinese musicians (6 males; 7 females; mean age ± SD: 25 ± 2.96 years) participated in the current study. All of the English and Chinese musicians received professional training (English mean years ± SD: 14.23 ± 4.60 years; Chinese mean years ± SD: 9.08 ± 3.93 years). Recruited participants signed the consent forms approved by the Internal Review Board (IRB) at University of Florida and the Human Subjects Ethics Sub-committee at the Hong Kong Polytechnic University. All participants were also compensated for participating in the study. No speaking, hearing or language difficulty were reported by the participants. None of the English participants received formal instructions in Mandarin Chinese or other tonal languages. To examine the effects of musical training, data obtained from musicians in this study were compared to those of non-musicians in Chen et al. (2017). Specifically, we recruited fifteen native Chinese speakers (mean age ± SD: 24.8 ± 2.83 years; 6 males; 9 females) from the Hong Kong Polytechnic University and fifteen native English speakers (mean age ± SD: 20.4 ± 1.96 years; 7males; 8 females) were recruited from the University of Florida to participate in that study.

### Stimuli

We recorded a male native Chinese speaker who has no speaking or hearing difficulty using an Audio-Technica AT2020 microphone in the phonetics lab at the University of Florida. Pitch contours on both low and high vowels ([a] and [i]) were then manipulated using the pitch synchronous overlap add (PSOLA) method [59] to generate all the stimuli. One level-to-rising continua and another level-to-falling continua were made, which consisted of seven-steps on both low vowel [a] and high vowel [i]. Also, we created nine sets of the two continua with different durations (from 200ms, 180ms, 160ms, 140ms, …, 40ms). Thus, we generated a total of 36 continua [2 (pitch directions) x 2 (vowels) x 9 (durations)], including 252 stimuli [7 steps * 36 continua].

In order to examine the relationship between speech production and perception, we generated all contour stimuli by manipulating the slope and intercept values of the linear pitch contour based on the estimation of underlying pitch targets [slope: 93.4; intercept: -2.2 semitones (st)] obtained from a corpus of real speech in Mandarin [60]. For example, to generate a rising continuum, we first calculated the offset F0 value for each duration. If the duration value was t = 200ms (0.2s), then the offset value in semitone was calculated in Eq (1).

X(t)=93.4*t−2.2=93.4*0.2−2.2=16.48st (1)

Then the obtained offset value in semitone (16.48st) was transformed to its corresponding value in Hertz with a baseline of 130Hz (Xu et al., 2006) using Eq (2), to obtain the offset F0 of 196.56 Hz.

$$Number\;of\;semitones=\frac{12}{{log}_{10}2}*log(F_{02}/F_{01})$$ (2)

**Fig 2:**
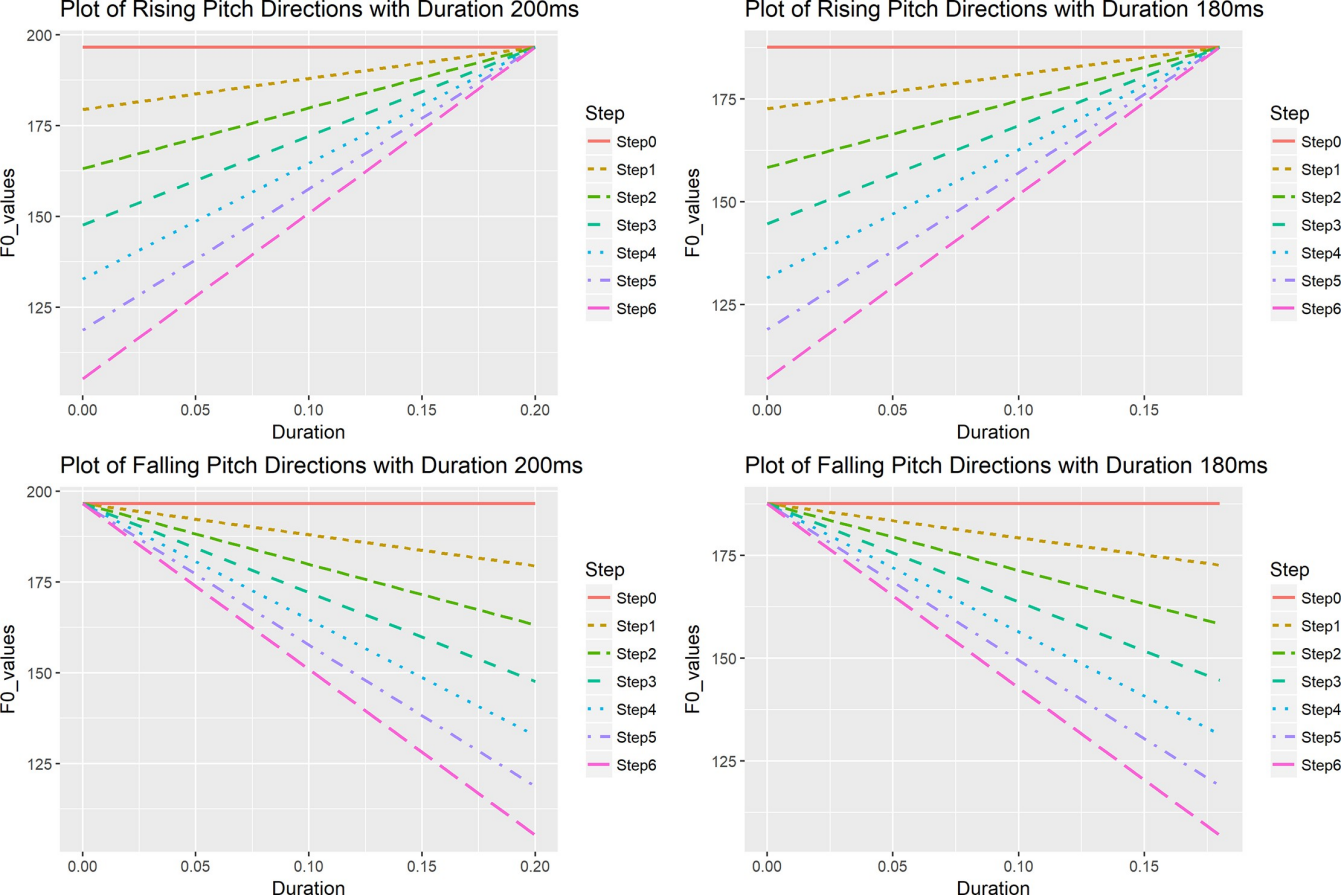
Rising and falling stimuli in two sets of durations 200ms and 180ms.

The onset was calculated by setting t = 0ms (s), which is -2.2st (123.02Hz). We then calculated the distance between onsets and offsets, which was 18.68st. Next, the onset value (-2.2st) was treated as the cutoff point and two onset values were defined, one as the low onset (LO), which was below -2.2st, and the other as the high onset (HO), which was above -2.2st. These two points were calculated so that the distance between LO and -2.2st was one third of the distance between HO and -2.2st. For stimulus duration of 200ms, LO was obtained by deducting 1/3*18.68st (onset-to-offset distance: 18.68st) from -2.2st, which was -8.43st (105.23Hz). HO was set to the same offset value of 196.56Hz to make it a level tone. We then created in-between steps on the ERB scale, which is a better scale for natural perception [39]. Thus, we created seven-step stimuli with equal perceptual distance for each continuum and the onset values were then transformed back into Hertz. The resynthesizing procedure involved adjusting the duration of the stimuli to nine duration values, peak normalizing the stimuli to the same intensity level, reducing the number of pitch points and adjusting them to desired values. For nine duration values, Tables 1 and 2 presented calculated onset or offset values for rising and falling pitch directions. Fig 2 presents the steps in falling and rising pitch directions with durations 200ms and 180ms. We created the falling pitch directions, where the order of the onset values was reversed to those of rising pitch directions. The stimuli were identical to those used in [47]. More details on stimulus generation procedure can be found in [47].

**Table 1:** Onset F0 values for each step of rising pitch continua with various duration.

Duration	0.2	0.18	0.16	0.14	0.12	0.1	0.08	0.06	0.04
**Onset 1**	105.23	106.89	108.57	110.28	112.01	113.78	115.57	117.39	119.24
**Onset 2**	118.67	118.92	119.21	119.55	119.92	120.33	120.79	121.28	121.82
**Onset 3**	132.77	131.49	130.27	129.13	128.05	127.04	126.11	125.24	124.43
**Onset 4**	147.58	144.61	141.76	139.03	136.41	133.91	131.52	129.24	127.06
**Onset 5**	163.12	158.31	153.69	149.26	145.02	140.94	137.04	133.30	129.72
**Onset 6**	179.43	172.62	166.09	159.84	153.86	148.14	142.66	137.42	132.40
**Onset 7**	196.56	187.56	178.97	170.78	162.96	155.50	148.38	141.59	135.11

**Table 2:** Offset values for each step of falling pitch continua with various duration.

Duration	0.2	0.18	0.16	0.14	0.12	0.1	0.08	0.06	0.04
**Offset 1**	196.56	187.56	178.97	170.78	162.96	155.50	148.38	141.59	135.11
**Offset 2**	179.43	172.62	166.09	159.84	153.86	148.14	142.66	137.42	132.40
**Offset 3**	163.12	158.31	153.69	149.26	145.02	140.94	137.04	133.30	129.72
**Offset 4**	147.58	144.61	141.76	139.03	136.41	133.91	131.52	129.24	127.06
**Offset 5**	132.77	131.49	130.27	129.13	128.05	127.04	126.11	125.24	124.43
**Offset 6**	118.67	118.92	119.21	119.55	119.92	120.33	120.79	121.28	121.82
**Offset 7**	105.23	106.89	108.57	110.28	112.01	113.78	115.57	117.39	119.24

We presented all re-synthesized stimuli in two tasks: an identification task and a same-different task. In the identification task, 1,260 stimuli were presented randomly (2 vowels * 9 duration * 7 steps * 2 pitch directions * 5 repetitions) in two blocks using the E-prime software, one for the falling continuum and one for rising continuum. In the same-different discrimination task, we paired all stimuli in each rising and falling continuum with a two-step difference, shown to be easier for listeners to perceive [61]. The stimuli include both pairs containing the same stimuli (0–0, 1–1, 2–2, etc.) and pairs containing different stimuli (0–2, 1–3, 2–4, 3–5, 4–6 or 2–0, 3–1, 4–2, 5–3, 6–4). We repeated all the stimuli twice and they were presented in a random order in two blocks using the E-prime software. In total, there were 1,224 trials (2 syllables*2 continua*9 durations*17 pairs). For both identification and same-different tasks, the order of all the blocks was also counterbalanced.

### Procedure

We used a GMH C 8.100 D head set for all native Chinese musicians and non-musicians participants in the current study at the speech lab of the Hong Kong Polytechnic University. We used a set of BOSE AE2 headphones for all English musicians and non-musicians participated at the phonetics lab of the University of Florida. Before experimental trials began, participants were first familiarized with level, rising, and falling pitch directions by listening to tones and looking at pictures of tonal contours, and familiarized with the experimental procedure. After hearing a stimulus in the ID task, participants made a judgment by pressing buttons representing a level tone and a rising/falling tone. After hearing a pair of two stimuli in the SD task, participants practiced pressing buttons to represent their choice of the stimuli being the same (number key 1) or different (number key 2). An ISI (interstimulus interval) of 500ms was chosen in this study, which may maximally distinguish the differences in between- vs. within- category discrimination [29]. The participants made judgments after hearing the stimuli at a self-paced rate. In prescreening, to ensure that they had the ability to identify or discriminate the stimuli and were familiar with the setting, we tested participants using stimuli of the biggest duration value of 200ms. All participants needed to pass a minimum threshold before recruitment to make sure their performance was above the chance level by a binomial test. The training was conducted to familiarize English musicians with Chinese syllables and to minimize differences in task difficulty [39]. We did not analyze the results from the practice session.

### Data analysis

#### Identification task

First, we used a generalized linear mixed model with a random effect of subjects using the “lme4” R package [62] to examine factors that might contribute to identification results, including stimulus duration, musical experience, pitch directions and vowel quality. To plot results for a comparison, we divided all stimuli into eight groups according to pitch directions, musical experience and vowel quality (FEMA, FEMI, REMA, REMI, FENA, FENI, RENA, RENI). The letters R and F stand for rising and falling pitch, EM and EN stand for English musician and English non-musicians, and A and I stand for low and high vowels. Similarly, there are eight groups for Mandarin musicians and non-musicians (FMMA, FMMI, RMMA, RMMI, FMNA, FMNI, RMNA, RMNI), MM and MN stand for Mandarin musician and Mandarin non-musicians.

Second, we calculated category boundary sharpness and the boundary location for each stimulus duration. In order to do so, we fit a generalized linear mixed effects model again for each subgroup. The response variable is identification scores (0 or 1) and the predictor is step number (x = 0–6). We used a logistic regression to model the relationship between identification and step number [39]. The fixed effect in our model is similar to a logistic regression model as in Eq (3), and we further added a random effect of subjects.

$${log}_{e}\left(\frac{p_{1}}{1-P_{1}}\right)=b_{0}+b_{1}x$$ (3)

In Eq (3), the coefficient *b*_*1*_ represents the sharpness of category boundary [39], and we extracted *b*_*1*_ from the fitted linear mixed effects models. To compare the identification in each pair of different stimulus duration, post-hoc analyses using a likelihood ratio test were conducted within each subgroup. Two models were fitted to each pair, where one model treated the coefficient *b*_*1*_ extracted from each pair as the same, and the other model treated them as different. A significant difference between the two models indicated that the coefficient *b*_*1*_ was different, and thus this was the difference in sharpness of category boundary between the pair. Similar post-hoc analyses were conducted on the effects of musical experience, pitch directions and vowel quality. We also examined the relationship between stimulus duration and category boundary sharpness for native English and Mandarin musicians.

In addition, we also obtained category boundary (*xcb*) for each subject, which was estimated using the proportion of -b_0_/b_1_ in Eq (3) [39] to obtain category boundary for each subject. Post-hoc analyses were also conducted to test for the effects of musical experience, pitch directions, duration and vowel quality on the location of category boundary. Again, we attempted to capture the relationship between duration and category boundary location for English and Mandarin musicians using regression models.

Finally, following [47], we obtained formulae of the time required for pitch perception. The formulae are presented in Eq (4) where *t* represents the required stimulus duration for perceiving *d* st differences (rising or falling) from level tones.

$$t=b_{0}+b_{1}d$$ (4)

We first estimated the stimulus step where the identification rate was 50%, which was set as a cut-off point because a step number smaller than this point meant that subjects tended to perceive it as a level tone. The cut-off step was the smallest step number where a pitch direction was perceived as different from a level one [47]. Since the formulae are to be compared with those derived from speech production results, they further transformed the cut-off step to st values. We proceeded to fit linear mixed effects models to obtain formulae that capture the stimulus duration needed for effective tone perception.

#### Same-different discrimination task

First, from the correct answer and the obtained responses, we calculated the correct (hits) and incorrect responses (false alarms), and computed A-prime scores in the same-different task [63, 64]. In order to examine the contributions of duration, musical experience, pitch directions and vowel quality, we fitted a generalized linear mixed model with all interaction terms to A-prime scores.

Second, *P*_*bc*_ (between-category discrimination) was defined as the comparison unit that corresponds to the category boundary. *P*_*wc*_ (within-category discrimination) was defined as *P*_*02*_ and *P*_*46*_, which are the two comparison units at the extremes of the continuum [29]. The A-prime score of between- and within- category discrimination was calculated.

We calculated A-prime scores for *P*_*bc*_ and *P*_*wc*_ for all duration values. After obtaining the A-prime scores, we fitted linear mixed-effects models to test whether musical experience, pitch directions, vowel quality, and stimulus duration significantly contributed to the A-prime scores in Mandarin and English listeners.

Third, the peakedness was defined as *P*_*bc*_ minus *P*_*wc*_, and we fitted linear models to examine contributing factors and conducted post-hoc analyses.

Finally, we compared the predicted discrimination score P* with the observed discrimination score *P*. The predicted score P* was defined in Eq (5):

$$P^{*}=[1+{\left(P_{A}-P_{B}\right)}^{2}]/2$$ (5)

where *P*_*A*_ and *P*_*B*_ stand for the identification scores [65]. We proceeded to calculate the correlation of P* and *P* and the mean difference between the two for each subgroup with each duration and tested whether some factors significantly contributed to the correlation.

## Results

### Identification task

#### Sharpness of category boundary

A generalized linear mixed effects model was fitted to the identification rate of both Mandarin and English speakers with and without musical experience. For English musicians and non-musicians, the results revealed significant or marginally significant main effects of the following variables: vowel quality [χ^2^ (1) = 43.46, p < 0.001], duration [χ^2^ (1) = 6341.9, p < 0.001] and pitch direction [χ^2^ (1) = 3.52, p = 0.06]. The two-way interaction between vowel and pitch direction [χ^2^(1) = 12.97, p < 0.001], vowel and duration [χ^2^(1) = 9.35, p = 0.002], music experience and duration [χ^2^(1) = 462.39, p < 0.001], and pitch direction and duration [χ^2^(1) = 135.35, p < 0.001] also reached significance. Neither of the three-way interactions reached significance.

**Fig 3:**
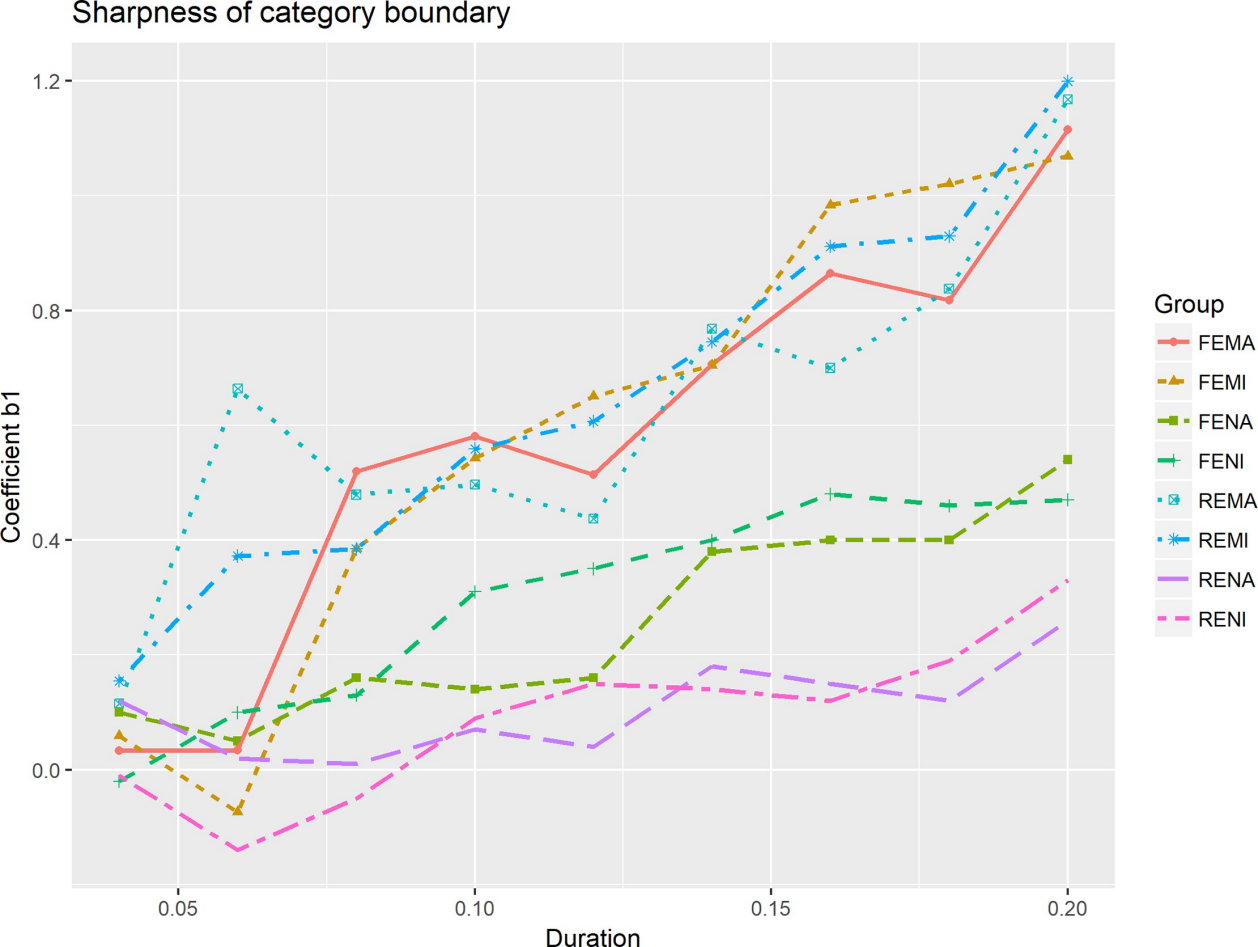
Sharpness of category boundary for English musicians and non-musicians.

**Fig 4:**
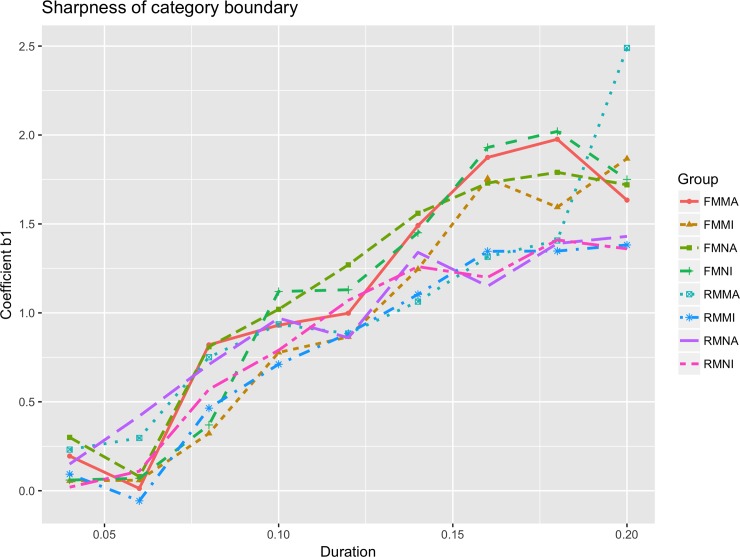
Sharpness of category boundary for Mandarin musicians and non-musicians.

From Fig 3, both English non-musicians and musicians also showed an increasing trend of sharpness of category boundary (coefficient b1) as stimulus duration increased. However, English musicians had sharper category boundaries than English non-musicians in most cases. The sharpness of category boundary for Mandarin musicians and non-musicians is shown in Fig 4. Yet, in comparison to English musicians and English non-musicians the differences in category boundary sharpness between Mandarin musicians and non-musicians is less discernible. We further modelled the change in sharpness and compared sharpness between Mandarin and English musicians and non-musicians.

Two regression models were fitted for English and Mandarin musicians to explore the relationship between stimulus duration and the category boundary sharpness. Compared to English non-musician [slope = 2.36], English musicians did show a steeper slope of 5.98, indicating a faster increment of sharpness of category boundary with increased duration.

With the increase of stimulus duration, category boundary sharpness also increased for both Mandarin musicians and non-musicians. Unlike Mandarin non-musicians, who showed a fast increase first and slowed down, the sharpness for Mandarin musicians increased linearly with a slope of 11.38. Also, Mandarin musicians have sharper category boundary for longer duration values 180ms and 200ms. For most duration values, the sharpness of category boundary can be ranked as English non-musician < English musicians < Mandarin musicians and non-musicians.

In sum, Mandarin musicians and non-musicians exhibited sharper category boundary than English musicians and non-musicians. However, while English musicians exhibited a significantly sharper category boundary than English non-musicians, the difference in category boundary sharpness between Mandarin musicians and non-musicians is non-significant. In addition, with the increase of stimulus duration, category boundary sharpness for both English musicians and non-musicians also increased, but sharpness of category boundary for English musicians increased faster. Unlike Mandarin non-musicians, Mandarin musicians exhibited a linear trend in the increasing rate.

#### Category boundary location

We fit regression models for English and Mandarin musicians to examine the relationship between stimulus duration and location of category boundary. For English musicians, a quadratic term was significantly different by a likelihood ratio test [χ^2^ (1) = 8.75, p = 0.003]. Specifically, Eq (6) presents the the formula of category boundary (*cb*) and stimulus duration (*d*).

$$cb=2158.59*d^{2}-735.9*d+64.34$$ (6)

English musicians perceived a category boundary earlier than English non-musicians for various durations, and the location of the category boundary decreased faster with increased duration than non-musicians in most cases.

For Mandarin musicians, a model with a slope and intercept differed significantly from a model with an extra quadratic form [χ^2^(1) = 15.25, p < 0.001]. Eq 7 lists the formula.

$$cb=244.43*d^{2}-77.39*d+8.99$$ (7)

Mandarin non-musicians tend to have earlier boundaries than Mandarin musicians for duration values less than 140ms. Both groups showed a generally earlier boundary location as the stimulus duration increased, and non-musicians decreased faster than musicians.

#### Perception formulae

Formulae for time required to produce rising and falling pitch directions by tonal and non-tonal speakers have been proposed by [58]. To make a comparison with formulae proposed for speech production, we also fitted linear regression models to capture the relationship between stimulus duration required for perception and pitch changes (rising or falling).

To explore the effects of musical experience, we compared the formulae obtained for each pitch direction for musicians to those obtained for non-musicians [47]. Again, *t* stand for the stimulus duration required to effectively perceive *d* st differences.

Rising,Englishmusicians:t=108.84+4.64*d (8)

Falling,Englishmusicians:t=101.97+5.16*d (9)

Rising,Englishnon‐musicians:t=137.33+1.89*d (10)

Falling,Englishnon‐musicians:t=144.66+1.5*d (11)

By likelihood ratio tests, English musicians and non-musicians showed different values of slope and intercept [χ^2^ (2) = 12.18, p = 0.002] with smaller intercepts and sharper slopes for English musicians. The intercept and slope values for falling and rising pitch also differed significantly [χ^2^ (2) = 10.81, p = 0.045]. Both intercept and slope values reached marginal significance for low and high vowels [χ^2^ (2) = 5.15, p = 0.076].

Moreover, based on the proposed formula, we specifically calculated the required time to perceive a 1 to 20 semitone change in pitch (rise or fall). Compared with formulae proposed for pitch production, the time required for perception was shorter than production when the rise was greater than 5st and the fall was greater than 3st. In addition, for English musicians, to perceive a falling pitch, a shorter duration was needed than a rising pitch direction for a rise or fall less than 13st.

Similarly, we obtained formulae for Mandarin musicians, and made a comparison to the formulae for non-musicians.

Rising,Mandarinmusicians:t=67.93+9*d (12)

Falling,Mandarinmusicians:t=64.48+8.11*d (13)

Rising,Mandarinnon‐musicians:t=66.76+8.39*d (14)

Falling,Mandarinnon‐musicians:t=99.45+5.29*d (15)

The intercept and slope values for falling and rising pitch differed significantly between musicians and non-musicians [χ^2^ (2) = 33.31, p < 0.001].

In addition, we calculated required time to effectively perceive a 1 to 20 semitone change in pitch. Again, the required time required for perception was shorter than that of production (if the fall was less than 16st). In addition, for Mandarin musicians, a falling pitch contour can be perceived within a shorter time than a rising one. The rank of the time required to perceive a rising pitch direction was MN < MM < EM < EN when the rise was less than 10st. The rank for a falling pitch direction was MM < MN < EM < EN when the fall was less than 12st.

### Same-different discrimination task

We fit generalized linear mixed effects models to test the significance of main effects and interaction terms. For English musicians and non-musicians, the main effects of the following variables reached significance: vowel quality [χ^2^ (1) = 6.00, p = 0.014], musical experience [χ^2^ (1) = 4.26, p = 0.04], pitch direction [χ^2^ (1) = 401.4, p < 0.001], and duration [χ^2^ (1) = 2472, p < 0.001]. The two-way interaction between musical experience and vowel quality [χ^2^ (2) = 7.78, p = 0.02], musical experience and duration [χ^2^ (1) = 9.15, p = 0.002], musical experience and pitch direction [χ^2^ (2) = 56.61, p < 0.001], and pitch direction and duration [χ^2^ (1) = 17.64, p < 0.01] reached significance. The three-way interaction did not reach significance.

For Mandarin musicians and non-musicians, the main effects of the following variable reached significance or marginal significance: vowel quality [χ^2^ (1) = 3.01, p = 0.083], pitch direction [χ^2^ (1) = 161.2, p < 0.001], and duration [χ^2^ (1) = 2664.5, p < 0.001]. The two-way interaction between musical experience and vowel quality [χ^2^ (2) = 15.16, p < 0.001], musical experience and duration [χ^2^ (1) = 5.43, p = 0.02], musical experience and pitch direction [χ^2^ (2) = 4.64, p = 0.098] reached significance or marginal significance. The three-way interaction among pitch direction, duration and musical experience [χ^2^ (2) = 5.52, p = 0.06] and among vowel quality, duration and musical experience [χ^2^(2) = 5.56, p = 0.06] reached significance.

#### Between-category discrimination

We fit linear mixed effects models with A-prime scores as the response variable, and musical experience, vowel quality, pitch directions, and duration as predictors. The subjects were then included as a random effect. For English musicians and non-musicians, the main effects of pitch direction [χ^2^ (1) = 27.42, p < 0.001] and musical experience [χ^2^ (1) = 5.42, p = 0.02] reached significance. The two-way interaction between musical experience and duration [χ^2^ (1) = 9.46, p = 0.009], pitch directions and duration [χ^2^ (1) = 16.40, p < 0.001], vowel quality and duration [χ^2^ (1) = 11.68, p = 0.003], were also significant. The three-way interaction of musical experience, duration and pitch directions [χ^2^ (1) = 6.58, p = 0.01] reached significance.

For Mandarin musicians and non-musicians, the main effects of the variables musical experience [χ^2^ (1) = 3.11, p = 0.078], duration [χ^2^ (1) = 222.66, p < 0.001] and pitch directions [χ^2^ (1) = 8.68, p = 0.003] reached significance or marginal significance by likelihood ratio tests. The two-way interaction term between duration and musical experience [χ^2^ (1) = 228.13, p < 0.001], pitch direction and musical experience [χ^2^ (1) = 16.70, p < 0.001], vowel quality and pitch directions [χ^2^ (1) = 5.78, p = 0.055], duration and pitch direction [χ^2^ (1) = 43.78, p < 0.001] reached significance. The three-way interaction of musical experience, duration and pitch direction [χ^2^ (1) = 76.94, p < 0.001], musical experience, vowel quality and duration [χ^2^ (2) = 5.11, p = 0.078] reached significance.

We fit a regression model for musicians and non-musicians of English and Mandarin speakers to explore the relationship between stimulus duration and A-prime scores. For English musicians, the duration significantly contributed to the A-prime scores [slope = 1.03; F (1, 258) = 8.77, p = 0.003; adjusted R^2^ = 0.029]. However, for native English non-musicians, duration did not reach significance [t (6) = 0.15, p = 0.88]. On average across all conditions, English musicians have higher A-prime scores (0.74) than non-musicians (0.64).

Similarly, we fit one regression model for Mandarin musicians and non-musicians. For Mandarin musicians, a quadratic term did not reach significance, as shown in a likelihood ratio test [χ^2^ (1) = 0.42, p = 0.52]. We thus fitted a simple linear regression instead and duration reached significance [F (1, 453) = 249.1, p < 0.001; adjusted R^2^ = 0.35].

Compared to Mandarin non-musicians (slope = 1.0), Mandarin musicians showed a steeper slope (10.61). Both groups showed increasingly better discrimination scores as the stimulus duration increased. On average, Mandarin non-musicians (0.59) showed lower between-category scores than Mandarin musicians (0.7).

In sum, the main effects of musical experience and pitch directions on characteristics of CP reached significance for both English and Mandarin musicians and non-musicians. A linear regression model for English musicians captures the relationship between A-prime scores and duration. However, for native English non-musicians, duration did not significantly contribute to the change in the A-prime scores in a regression model. Compared to Mandarin non-musicians, Mandarin musicians showed a steeper slope, indicating higher discrimination scores as the stimulus duration increased.

#### Within-category discrimination

For English musicians and non-musicians, we fit a linear mixed effects model. The main effects of the following variables reached significance: pitch direction [χ^2^ (1) = 4.55, p = 0.03], musical experience [χ^2^ (1) = 70.73, p < 0.001], vowel quality [χ^2^ (1) = 9.46, p = 0.002] and duration [χ^2^ (1) = 27.04, p < 0.001]. The two-way interaction term between musical experience and pitch directions [χ^2^ (1) = 7.3, p = 0.007], pitch directions and duration [χ^2^ (1) = 60.53, p < 0.001], musical experience and duration [χ^2^ (1) = 111.96, p < 0.001], musical experience and vowel quality [χ^2^ (1) = 13.43, p < 0.001] reached significance. The three-way interaction of musical experience, duration and pitch direction [χ^2^ (1) = 30.637, p < 0.001], musical experience, vowel quality and duration [χ^2^ (1) = 31.58, p < 0.001], pitch direction, musical experience and duration [χ^2^ (1) = 9.14, p = 0.03] reached significance.

For Mandarin speakers, the variables pitch direction [χ^2^ (1) = 40.09, p < 0.001], musical experience [χ^2^ (1) = 48.02, p < 0.001], vowel quality [χ^2^ (1) = 19.08, p < 0.001] and duration [χ^2^ (1) = 123.98, p < 0.001] significantly contributed to within-category discrimination. The two-way interaction of pitch direction and vowel quality [χ^2^ (1) = 12.37, p < 0.001], pitch direction and duration [χ^2^ (1) = 4.44, p = 0.035], musical experience and duration [χ^2^ (1) = 14.45, p < 0.001] were also significant. The three-way interaction terms musical experience, duration and pitch direction [χ^2^ (1) = 5.87, p = 0.015] reached significance.

On average, English musicians (0.81) have higher within-category A-prime scores than English non-musicians (0.44). Mandarin musicians showed higher within-category discrimination scores (0.67) than non-musicians (0.48) on average.

#### Peakedness

The variables that significantly contributed to peakedness include pitch direction [χ^2^ (1) = 3.95, p = 0.047] and duration [χ^2^ (1) = 4.72, p = 0.03] were significant. Musical experience [χ^2^ (1) = 3.25, p = 0.071] reached marginal significance. The interaction term between duration and pitch direction [χ^2^ (1) = 7.81, p = 0.005] also reached significant.

For Mandarin musicians and non-musicians, the main effects of pitch direction [χ^2^ (1) = 26.51, p < 0.001] and duration [χ^2^ (1) = 3.42, p = 0.06] reached significance or marginal significance. The two-way interaction of musical experience and duration [χ^2^ (1) = 7.57, p = 0.0059], musical experience and pitch direction [χ^2^ (1) = 10.67, p = 0.0048], pitch direction and vowel quality [χ^2^ (1) = 31.64, p < 0.001]. The three-way interaction term of duration, pitch direction and musical experience [χ^2^ (1) = 11.71, p = 0.0029], duration, pitch direction and vowel quality [χ^2^ (1) = 7.67, 0.053], pitch direction, musical experience and vowel quality [χ^2^ (1) = 5.40, p = 0.067] reached significance or marginal significance.

#### Predicted and obtained discrimination

For the correlation between obtained and predicted discrimination in English musicians and non-musicians, we fitted a linear model. The main effects of pitch direction [t(125) = 7.66, p < 0.001] and the two-way interaction between musical experience and pitch direction [t(125) = -5.13, p < 0.001], between pitch direction and vowel [t(125) = -2.47, p = 0.015], between musical experience and duration [t(125) = 2.16, p = 0.033] and between pitch direction and duration [t (125) = -6.955, p <0.001] reached significance. The three-way interaction among musical experience, pitch direction and duration was also significant [t (125) = 3.737, p < 0.001]. The three-way interaction between musical experience, pitch direction and vowel [t (125) = 1.80, p = 0.07], musical experience, vowel and duration [t (125) = -1.90, p = 0.059], as well as pitch direction, vowel and duration [t (125) = 1.73, p = 0.086] reached marginal significance.

Similarly, for Mandarin musicians and non-musicians, the main effect of vowel quality [t (343) = 2.29, p = 0.023], duration [t (343) = 2.89, p = 0.04] and the two-way interaction between pitch direction and musical experience [t (343) = 2.01, p = 0.0045], between pitch direction and vowel [t (343) = -2.26, p = 0.025], between vowel and duration [t (343) = -2.08, p = 0.038] also reached significance. The three-way interaction among vowel, direction and duration was marginally significant [t (343) = 1.91, p = 0.0057].

Finally, for English musicians and non-musicians, a linear model with the dependent variable of the distance P-P* suggested that the main effects of musical experience [t (135) = 4.93, p < 0.001], pitch direction [t (135) = -2.76, p = 0.0065], and duration [t (135) = 3.04, p = 0.0028] and the two-way interaction between pitch direction and musical experience [t (135) = -1.88, p = 0.063] was marginally significant.

**Fig 5:**
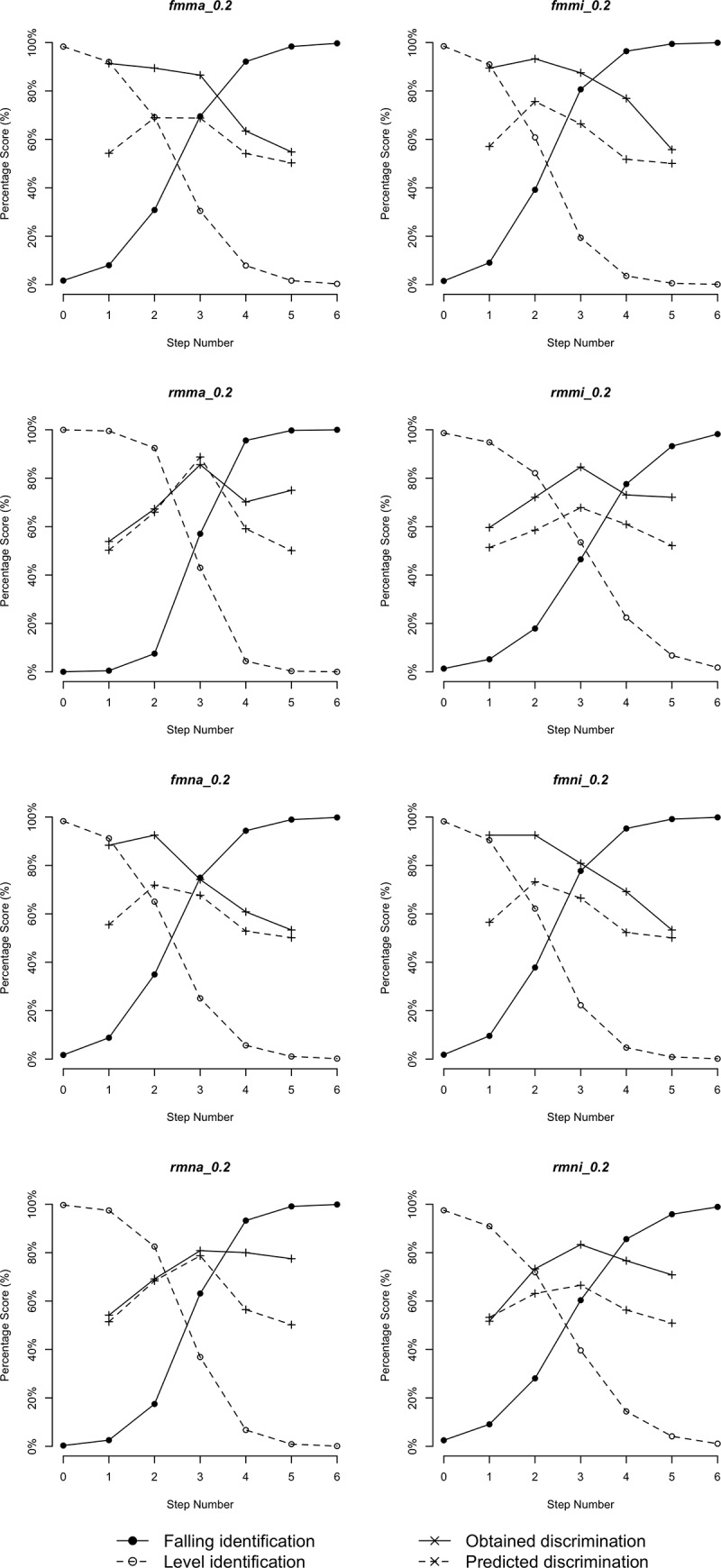
Identification and discrimination of stimuli with 200ms for Mandarin speakers.

**Fig 6:**
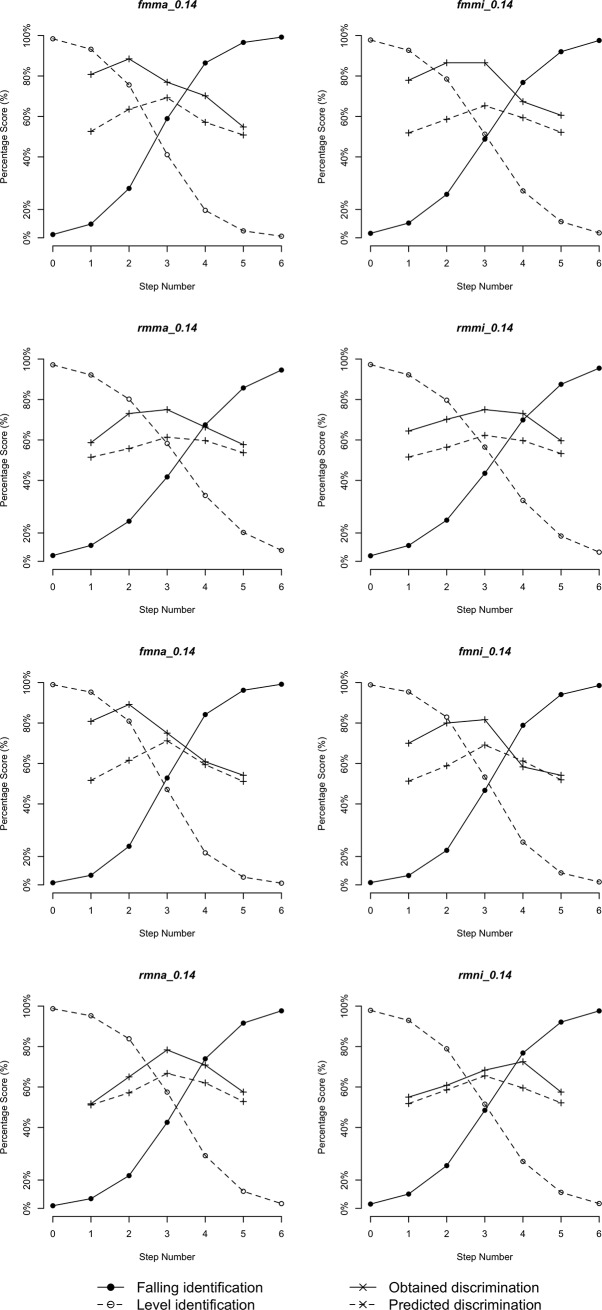
Identification and discrimination of stimuli with 140ms for Mandarin speakers.

**Fig 7:**
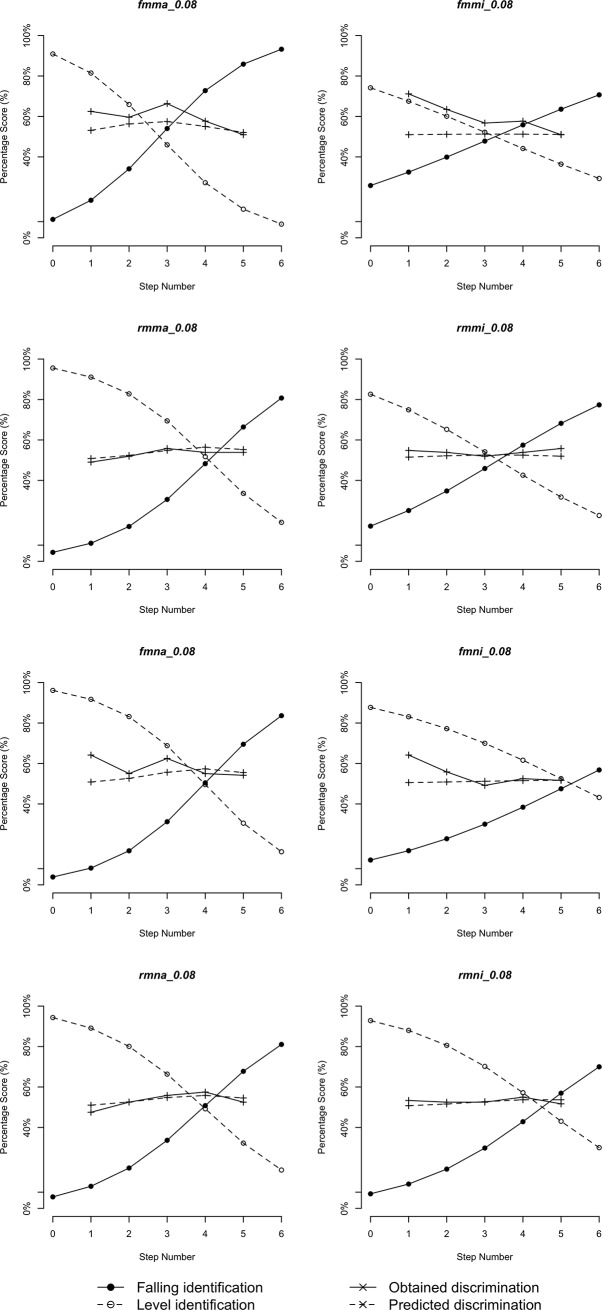
Identification and discrimination of stimuli with 80ms for Mandarin speakers.

**Fig 8:**
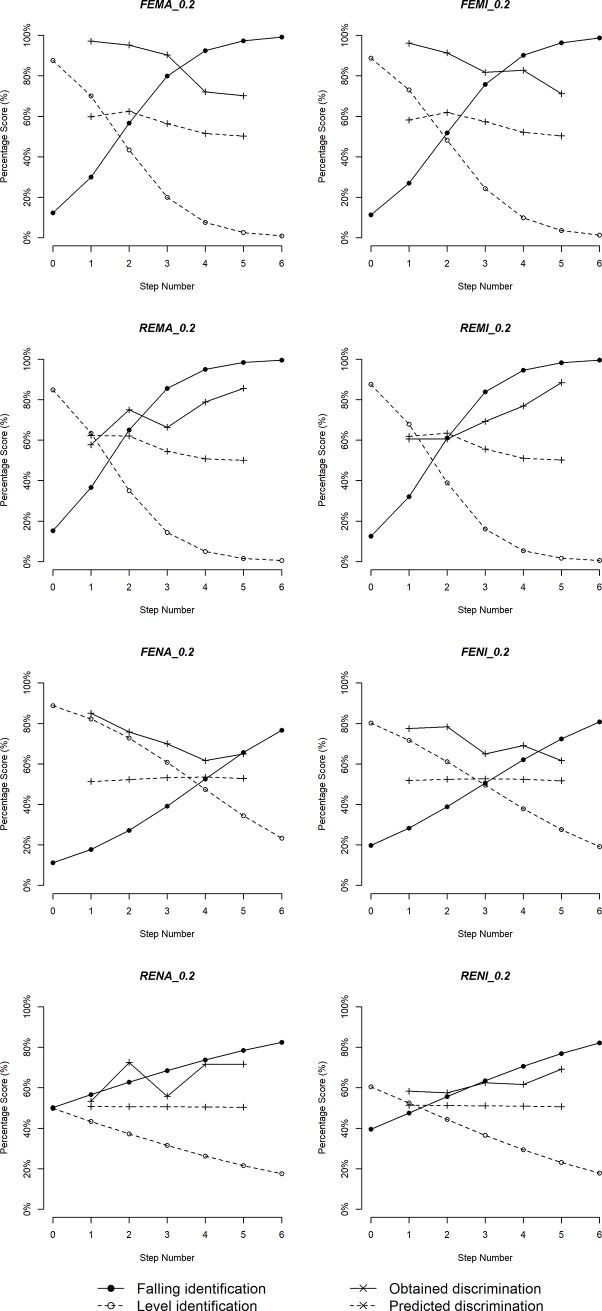
Identification and discrimination of stimuli with 200ms for English speakers.

**Fig 9:**
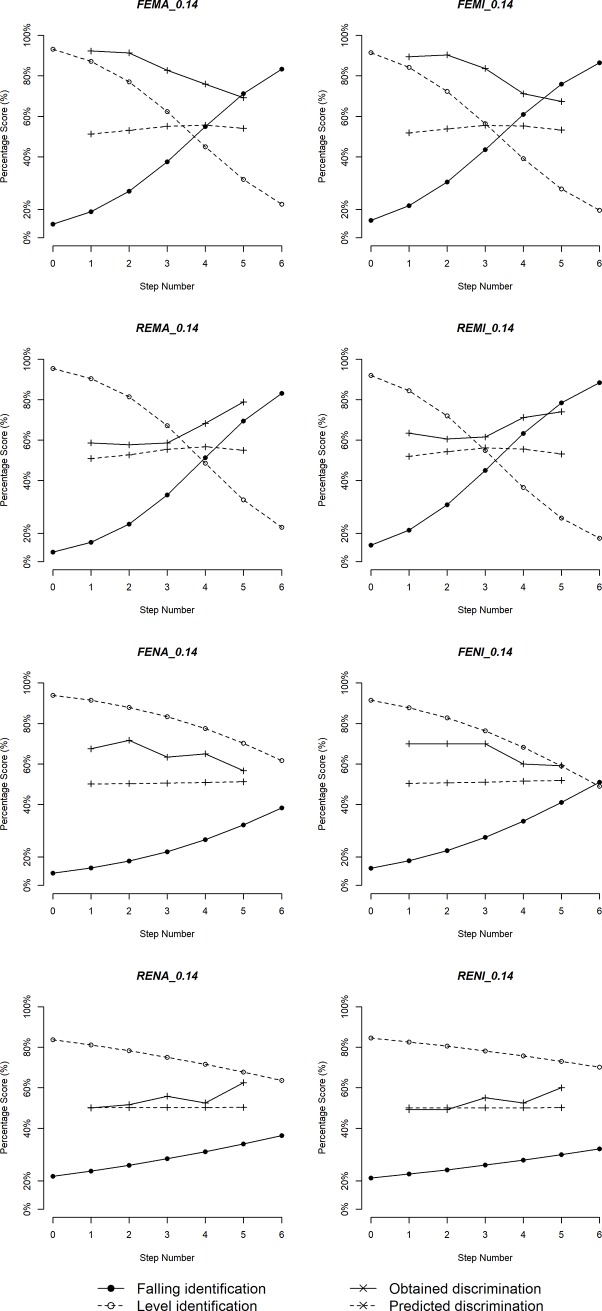
Identification and discrimination of stimuli with 140ms for English speakers.

**Fig 10:**
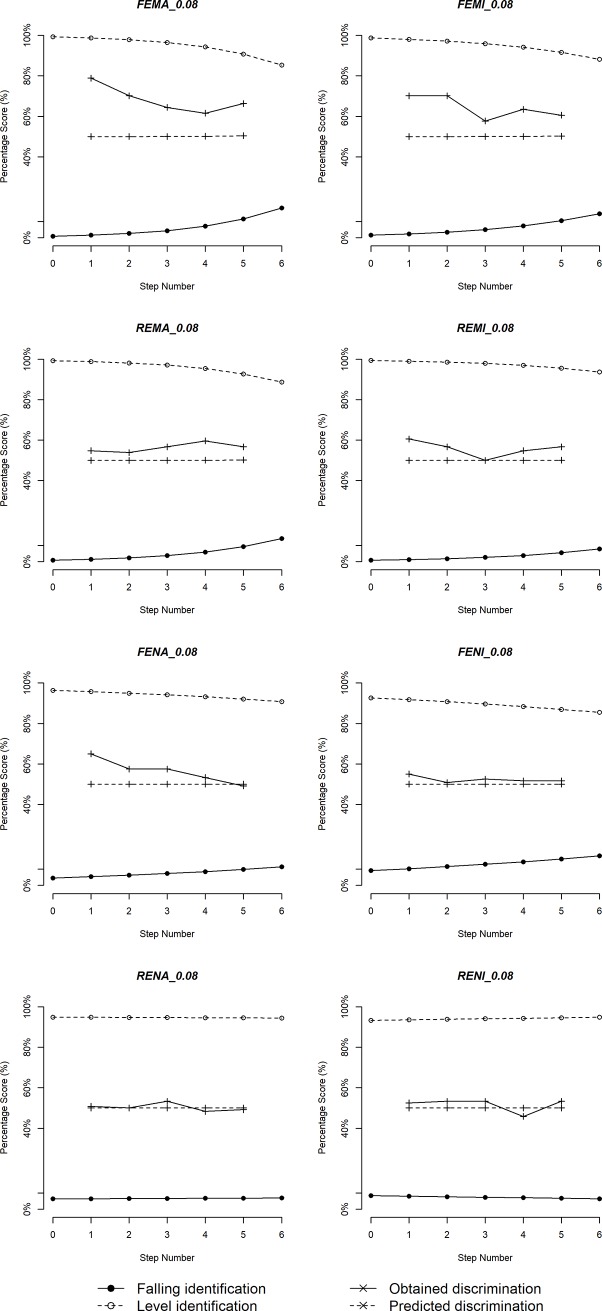
Identification and discrimination of stimuli with 80ms for English speakers.

Figs 5–10 plot identification functions and obtained vs. predicted discrimination of Mandarin and English musicians vs. non-musicians with three duration values as an example. From the figures, Mandarin musicians and non-musicians are similar in the sharpness of boundary. However, English musicians have significantly sharper boundary than non-musicians. The sharpness of category boundary increases as the duration of stimuli becomes longer, and the categorical perception becomes stronger for both musicians and non-musicians.

## Discussion

### Improved usage of stimulus duration, sensitivity to intrinsic f0 and pitch direction

Both English and Mandarin musicians exhibited an increase in the category boundary sharpness as stimulus duration increased. English musicians’ category boundary sharpness increased faster with increasing duration than English non-musicians. However, Mandarin non-musician’s sharpness increased faster than Mandarin musicians initially and then reduced to a slower rate with increasing stimulus duration. Moreover, from the results of the same-different task, both English and Mandarin musicians’ between-category sensitivities increased with the increment of stimulus duration, with a greater benefit realized by musicians than non-musicians in general.

Our results that categorical perception becomes stronger with longer duration among English and Mandarin musicians and non-musicians is inconsistent with the cue-duration hypothesis [29]. The hypothesis proposes that short acoustic cues with weaker auditory memory representations may lead to stronger categorical perception. For example, stop consonants are more categorically perceived than vowels because formant transitions, an acoustic cue to stop consonant distinction are shorter than vowel formants, acoustic cue to vowel distinction [25, 66–68]. However, cue-duration hypothesis cannot effectively account for categorical perception of pitch directions by both musicians and non-musicians speaking tonal and non-tonal languages. Our results revealed the opposite relationship between duration and strength of CP on pitch perception in both Mandarin and English musicians. In addition, both groups also exhibited a faster rate of increment in category boundary sharpness for longer duration values, suggesting that musicians benefitted more from duration increment in categorical perception than non-musicians. These results confirmed Bidelman’s predictions on the benefits of musical training in categorical perception of speech sounds [15]. Specifically, it is proposed that musicians are better at forming auditory representation for speech signals and their sound-to-internalized memory templates matching ability is relatively enhanced [15]. Although English musicians without exposure to tonal languages should not have tonal categories stored in their long-term memory, they still benefited more from increased stimulus duration than English non-musicians, probably due to their greater sensitivity to and better usage of the extra time to form a better auditory representation of sounds and match them to recently internalized memory templates. Tonal musicians with long-term phonemic memory of lexical tone categories may further benefit from the extra time in longer stimuli for context-coding, including a superior capability of matching features to long-term representations.

In addition, vowel quality significantly contributed to tone identification and sharpness of category boundary in English and Mandarin musicians. Vowel quality also plays a role in determining the time required for tone perception by both groups of listeners. For English musicians, the minimal duration needed to perceive tones in low and high vowels was significantly different, but no significant differences were found among English non-musicians. However, both Mandarin musicians and non-musicians showed significant differences in the duration needed to perceive pitch direction on low vs. high vowels. Moreover, in our study, unlike English and Mandarin non-musicians [47], vowel quality was found to contribute significantly to sharpness of category boundary in English and Mandarin musicians. Interaction terms involving vowel quality also reached significance in between- and within-category discrimination. Therefore, musicians excel not only at separating acoustic cues in order to use the most relevant cue (f0) in pitch perception [57], but also at teasing apart factors such as vowel quality that may affect the f0 cue.

Based on formulae proposed in Section 3.1.3, both rising and falling pitch directions on high vowels required less time to be accurately perceived by English musicians. However, vowel quality did not consistently affect perception by English non-musicians. English non-musicians required a longer duration to perceive rising tones on high vowels than on the low vowels (rise < 16st), but a shorter duration was needed to perceive a falling tone on high vowels than low vowel. Therefore, situations (1c) and (2b) depicted in Fig 1 capture the English musicians’ results and situations (1c) and 2(c) are consistent with the English non-musicians’ patterns. Mandarin musicians required a longer duration to perceive rising pitch contours on a low vowel [a] than on a high vowel [i] if the rise was greater than 9st, and a longer duration to perceive falling pitch contours on low vowel than high vowel if the fall was greater than 12st. Mandarin non-musicians required a longer duration to perceive rising tones on a low vowel than on a high vowel when the rise was greater than 10st, and longer duration for perceiving a falling tone on a low vowel if the fall was greater than 9st. Therefore, situations (1c) and (2b) in Fig 1 describe Mandarin musicians and non-musicians’ behavior within the above reported st range. Our results thus suggested that musicians did exhibit a stronger influence from intrinsic F0 effects in pitch perception, which was likely due to their greater sensitivity to pitch differences accompanying vowel quality difference in speech production in comparison to non-musicians, and in turn they compensated more for intrinsic F0 effects in pitch perception.

Finally, types of pitch directions (rising vs. falling) significantly contributed to the sharpness of category boundary in both English and Mandarin musicians. Based on the formulae proposed separately for rising and falling pitch directions, English non-musicians did not differ significantly in the time needed to perceive falling vs. rising pitch directions, but English musicians showed significant differences. Mandarin musicians showed greater differences in perceiving falling vs. rising pitch directions on both low and high vowels, but Mandarin non-musicians only showed significant differences on high vowels, indicating that they are less sensitive to differences in types of pitch directions. Our results showed that musicians generally exhibited greater differences in the time needed to perceive rising vs. falling pitch directions than non-musicians. In addition, both English and Mandarin musicians and non-musicians required a shorter duration to perceive a falling pitch than a rising one (if the rise and fall were less than 13st for English musicians), indicating no perceptual compensation mechanism involved since rising tones also require longer time to produce. It also confirms the findings reported in [69] that English words produced with a falling F0 are processed faster than rising F0 by native English and Chinese speakers. Therefore, considering all the above results and that musicians are more efficient in pitch processing, as illustrated in Section 4.3, musicians are more sensitive to differences in pitch directions. Furthermore, in comparison to non-musicians, the time musicians needed to perceive a falling pitch direction is much shorter than the time needed to perceive a rising one, which suggest that psychoacoustically, a falling pitch contour might be more salient than a rising one, particularly among musicians, whose sensitivity to small differences in pitch lead to a more exaggerated effect in the time difference in perceiving the two pitch types.

In sum, previous studies have shown that musical training enhances auditory processing of fundamental frequency and the enhancement can be transferred to speech processing, especially pitch processing in lexical tones [70]. Musical training also enhances neural sensitivities to both changes in musical pitch and speech in children, supported by improvement in word discrimination and enhanced positive mismatch responses to lexical tones [71]. Our results further revealed that musicians are also more likely to benefit more from increasing stimulus duration in processing pitch direction changes. In addition, musicians compensate more for the effect of intrinsic f0 in speech perception and are more sensitive to the differences in types of pitch directions, probably due to their greater sensitivity to inherent differences in pitch associated with vowel quality and psychoacoustical difference between the two types of pitch directions.

### Categorical perception by tonal and non-tonal musicians vs. non-musicians

In the identification task, we found that musical experience significantly increased the sharpness of category boundary in the perception of rising and falling pitch directions by both English and Mandarin speakers. However, category boundary was less sharp for English musicians than for native Mandarin musicians or non-musicians. Moreover, Mandarin musicians showed sharper category boundary for longer stimuli (180ms and 200ms) than Mandarin non-musicians. English and Mandarin musicians also showed earlier category boundary than non-musicians in general.

In the same-different tasks, musical experience contributed significantly to between-category A-prime scores in both English and Mandarin groups. English and Mandarin musicians showed higher between-category A-prime scores than English non-musicians. English and Mandarin musicians also had higher within-category A-prime scores than non-musicians. For peakedness, musical experience only reached marginal significance for English musicians compared to English non-musicians. Musical experience was not a significant main effect but interacted with stimulus duration and pitch directions for Mandarin speakers. Overall, English and Mandarin musicians showed stronger categorical perception than English and Mandarin non-musicians. However, in identification tasks, Mandarin musicians did not show consistently stronger categorical perception than Mandarin non-musicians.

The results for English speakers were inconsistent with the findings reported in [9], where no significant differences were found between English musicians and non-musicians in perceiving a Mandarin tone continuum. It might be that the benefit from musical experience is realized in categorical perception of only certain tonal types, such as rising and falling pitch directions in our study. As reported in [10] (T2-T3 continuum) and [51] (T1-T4 continuum), Mandarin musicians did not consistently perceive the pitch direction more categorically than non-musicians, suggesting a limit in the extent to which musical training can further strengthen an already strong categorical perception of native tones. However, both English and Mandarin musicians were more sensitive to within-category pitch changes, confirming enhanced pitch sensitivity due to musical training [51].

### Efficiency of tone perception by tonal and non-tonal musicians

Musicians usually exhibit greater precision in processing both musical pitch and fundamental frequency in speech. Our study examined whether higher degree of auditory precision in pitch processing by musicians would shorten the time required for categorization of pitch directions in the language domain or whether increased sensitivity to pitch of lexical tones is at the cost of slower processing.

We found that English musicians needed less time to process rising and falling pitch directions than English non-musicians when the rise was less than 11 st, and the fall was less than 12 st. The formulae for English musicians had significantly sharper slopes, suggesting that musical ability heightens musicians’ sensitivity to pitch changes and improves processing efficiency within a pitch change of a 12 st. Based on the proposed formulae capturing the relationship between pitch changes and time for efficient processing of pitch changes, Mandarin musicians needed more time than Mandarin non-musicians to process rising pitch directions, but not to process falling pitch directions. However, the intercept and slope values of the proposed formulae for Mandarin musicians and non-musicians did not differ significantly. We propose a ranking for the four groups in their required time to perceive a rising pitch direction as follows: MN < MM < EM < EN (the rise < 10st), and a falling pitch direction: MM < MN < EM < EN (the fall < 12st). Although musical experience improves the efficiency of English speakers in pitch processing within a pitch range less than 12st, it did not improve significantly and consistently for Mandarin speakers, which may be due to a ceiling effect of Mandarin speakers’ already high efficiency in pitch processing. English speakers were in general slower in processing pitch than Mandarin speakers regardless of their musical experience. After comparing the minimal duration required to produce and perceive pitch directions, our results showed that the minimum time required to “perceive” pitch directions by musicians was usually shorter than the required time to “produce” pitch directions, similar to the findings reported by [47] on non-musicians. Our results thus further confirmed that physical constraints affect speech production to a greater extent than perceptual constraints do on speech perception [72].

In sum, in most cases, musical training may lead to shorter processing time. The reasons may lie in the increased efficiency in attentional control network, where musicians can respond faster and more accurate when distracting information is present [73]. In the present study, musicians may be less disrupted by other irrelevant acoustic cues in identifying and discriminating tones, which leads to higher efficiency and less time required for tone perception. It has also been shown that musical training may lead to improved working memory in the auditory domain [74]. Updating auditory working memory is less effortful among musicians, hence they may process the incoming lexical tones more effectively.

## Conclusions

This study investigates the effects of musical training in improving efficiency of pitch processing, strengthening categorical perception and enhancing factors interacting with pitch processing. First, we found that non-tonal musicians showed stronger categorical perception of pitch directions, whereas tonal musicians did not consistently show the same pattern. Second, stimulus duration affects both tonal and non-tonal musicians, and they tend to be more efficient in pitch processing than non-musicians. Musicians also tend to benefit more from increasing stimulus duration in processing pitch changes, which may be due to their greater sensitivity to temporal information and their more efficient use of additional time to form a better auditory representation, matching sounds to internalized memory templates and context-coding, including greater ability in matching features to long-term representations. Third, musicians compensated more for intrinsic F0 in pitch perception due perhaps to their greater sensitivity to pitch differences. Finally, musicians exhibited greater sensitivity to differences in pitch direction types than non-musicians, and they needed less time to perceive a falling pitch direction than a rising one, which may indicate that falling pitch sounds more salient to musicians and is thus processed faster. These findings deepen our understanding factors affecting pitch perception including stimulus property, native experience with lexical tones and musical ability, and form the basis for a better understanding of how pitch is processed among special population. For example, it has been reported that Mandarin speaking children and adolescents with Autism Spectrum Disorder (ASD) have enhanced pitch processing of non-speech stimuli (melodic contour), but show deficits in pitch processing of speech stimuli (question vs statement intonation) [75]. This finding suggests that linguistic and non-linguistic (musical) pitch is processed differently and that native experience with lexical tones does not compensate for linguistic pitch processing deficits among individuals diagnosed with ASD. Enhanced processing of non-speech stimuli among ASD patients has also been reported in a neurophysiological (ERP) study by [76]. In this study, Mandarin children with ASD exhibited a deficit in categorical perception of Mandarin tones showing comparable neural sensitivity to between- and within-category deviants whereas enhanced neural sensitivity to between-category relatively to within-category deviant was observed among typically developed Mandarin children controls. Relatively greater sensitivity to within-category deviant among ASD children may have been due to their enhanced sensitivity to the acoustic rather than the linguistic features of the stimulus at the pre-attentive level [76]. However, unlike our adult musicians in the study, auditory sensitivity to the acoustic properties of the stimuli among ASD children appear to interfere with their ability to process them at a higher, more abstract level (i.e., categorization) of pitch processing. Further research is needed to confirm this hypothesis.

Acoustic fundamental frequency (f0) or its auditory impression of pitch is a common perceptual object in both music and language. Associations between musical ability and accuracy at perceiving lexical tone contrasts have been reported in previous investigations [77], which suggests that musicians are better than non-musicians in their ability to identify and discriminate lexical tones. Musically adapted social stories can modify behaviours in students with autism and enhance speech production (Brownell, 2002). If musical training is found to improve the use of speech prosody, new knowledge will be generated concerning the possibility of domain transfer in processing acoustic cues, such as pitch and intensity, from non-speech stimuli to speech stimuli, and the benefits of musical training in improving the use of acoustic cues in speech prosody. Future work is motivated to examine whether musical training may improve pitch processing ability and help listeners attend more to acoustic details in speech and improve categorical perception strength, especially in populations with speech disorders.

## Supporting information

S1 Raw Data English musicians. (ZIP)

S2 Raw Data Mandarin musicians. (ZIP)
